# Coupling of potential habitat models with particle tracking experiments to examine larval fish dispersal and connectivity in deep water regions

**DOI:** 10.1371/journal.pone.0308357

**Published:** 2024-08-12

**Authors:** Gonzalo Daudén-Bengoa, Julio Sheinbaum, Javier RodríguezOuterelo, Sharon Z. Herzka

**Affiliations:** 1 Departamento de Oceanografía Biológica, Centro de Investigación Científica y de Educación Superior de Ensenada (CICESE), Ensenada, Baja California, México; 2 Departamento de Oceanografía Física, Centro de Investigación Científica y de Educación Superior de Ensenada (CICESE), Ensenada, Baja California, México; Texas A&M University, UNITED STATES OF AMERICA

## Abstract

Computing Lagrangian trajectories with ocean circulation models is a powerful way to infer larval dispersal pathways and connectivity. Defining release areas and timing of particles to represent larval habitat realistically is critical to obtaining representative dispersal pathways. However, it is challenging due to spatial and temporal variability in larval density. Forward-tracking particle experiments were conducted to study larval connectivity of four species (neritic or mesopelagic) in the Gulf of Mexico’s (GoM) deep-water region. A seasonal climatology coupled with predicted potential larval habitat models based on generalized additive models was used to delimit the particle dispersal origin. Two contrasting mesoscale circulation patterns were examined: (1) high Loop Current (LC) intrusion, absence of recently detached LC anticyclonic eddies (LC-ACE), and no interaction between LC-ACEs and the semi-permanent cyclonic eddy (CE) in the Bay of Campeche (BoC), and (2) limited LC intrusion, a recently detached LC-ACE, and interaction between LC-ACEs and the BoC’s CE. To simulate larval transport, virtual larvae were randomly released in the potential habitats and advected for 30 days with the velocity fields of the HYbrid Coordinate Ocean Model with hourly-resolution assimilation. Potential habitat location and size played a major role in dispersal and connectivity. A greater percentage of particles were retained in potential habitats restricted to the southern BoC, suggesting lower connectivity with other GoM regions than those encompassing most of the BoC or the central Gulf. Mesoscale feature interactions in the western GoM and BoC led to greater dispersion along the western basin. By contrast, the absence of ACE-CE interaction in the BoC led to greater retention and less connectivity between the southern and northern GoM. Under high LC intrusion, particles seeded north of the Yucatan Shelf were advected through the Florida Straits and dispersed within the GoM. Coupling potential habitat models with particle experiments can help characterize the dispersal and connectivity of fish larvae in oceanic systems.

## Introduction

Marine fishes have complex life cycles in which each developmental stage may occupy habitat types with distinct environmental conditions. While larval habitat depends on adult distribution and spawning [1], the limited swimming capacity and small size of fish larvae imply that they can be dispersed over long distances to areas that are favorable or unfavorable to survival and recruitment [2, 3]. The small larval size relative to the scales of ocean circulation makes tracking individuals challenging, requiring multiple and interdisciplinary approaches [4]. Simulations of transport with ocean circulation models are conducive to estimating the time between spawning and recruitment to nursery habitats [5], identifying larval origin [6], and inferring retention areas [7]. Retention and dispersal are key factors determining the connectivity level between regions and the environmental conditions larvae experience [8, 9]. Evaluating the connectivity mediated by ocean currents and understanding the underlying transport processes is critical since it is linked to recruitment variability [9, 10], and it allows for inferences regarding population structure and the potential for recolonization following disturbances such as oil spills or hurricanes [11].

Since the horizontal swimming abilities of fish larvae are largely limited relative to the velocities of ocean currents, they can be transported over large distances, although active vertical positioning in the water column can also influence a larva’s dispersal distance and destination. Tracking fish larvae, inferring dispersal pathways, and evaluating connectivity in the open ocean is challenging due to their small size, requiring an interdisciplinary approach [4]. Numerical circulation models have provided insight into larval dispersion [10, 12], natal origin, and transport mechanisms by allowing for the reconstruction of dispersal pathways [6, 7]. These studies use Lagrangian particle-tracking frameworks coupled with ocean circulation models to identify the transport pathways of many individuals (virtual fish larvae) through space and time [13, 14]. Additionally, studies can integrate ecological or life history characteristics such as adult spawning habitat and seasonality, planktonic stage duration, larval growth rates, or vertical position in the water column, allowing for a more realistic evaluation of the larval dispersion [9, 15, 16].

The high temporal and spatial variability typically observed in the distribution and density of fish larvae in pelagic habitats makes deciding where to seed particles to obtain realistic dispersal pathways challenging. Potential habitats, which can be stage-specific, can be delimited based on a suitable set of environmental conditions in time and space [17] based on empirical relationships between density, location, and environmental conditions using statistical approaches such as generalized additive models (GAMs) [11, 18]. GAMs, an extension of generalized linear models, are nonparametric and nonlinear regression techniques that do not require a priori specifications of the functional relationship between the response and predictor variables [19, 20]. This approach to predicting pelagic larval habitat has been used to assess the impacts of anthropogenic disturbances on fish larvae, including climate change [17, 21, 22].

In the GoM’s deep-water region, the main oceanographic feature that modulates the circulation is the Loop Current (LC), which exhibits different degrees of intrusion [23–25]. During high LC intrusion, LC anticyclonic eddies (LC-ACE) can detach and travel westward over 6 to 11 months, gradually weakening and losing retentive capacity before interacting with the slope and dissipating [24, 26, 27]. The LC and LC-ACE influence both the retention and transport of water masses and pelagic plankton from the Caribbean Sea to the GoM as well as their transport through the Florida Strait [23, 28, 29]. Along the western GoM’s shelf, there is on-shelf and off-shelf transport when mesoscale eddies reach the slope and interact with along-shelf currents [30, 31]. The arrival of ACEs to the GoM’s western slope has been shown to transport the larvae of coastal or neritic fish species offshore in the western Gulf [6].

The southern GoM (sGoM), or Bay of Campeche (BoC; south of 22 ºN) has a semi-permanent cyclonic eddy (CE) on its western side that provides nutrients to the euphotic layer due to a shallowing of the pycnocline [32, 33]. The CE interacts with the slope and shelf, leading to the cross-shelf exchange of water masses [31]. Espinoza-Fuentes and Flores-Coto [34], Flores-Coto et al. [35] and Echeverri et al. [36] documented the presence of fish larvae of coastal and neritic species in the deep water region of the BoC, which indicates that offshore transport is a recurrent phenomenon, albeit with unknown consequences for recruitment. Wind-driven upwelling in the southeastern BoC near the Yucatan shelf (YS) and river runoff from the Grijalva-Usumacinta river system transported toward the deep waters of the GoM lead to offshore transport as well as higher productivity in the BoC than in the central gulf [30, 37–39].

The topography in the western BoC (wBoc) constrains the position and structure of the CE; however, its shape and intensity are influenced by its interaction with LC-ACEs or other mesoscale eddies [40, 41]. Perez-Brunius et al. [33] analyzed surface drifter data and documented northward jets in the central and eastern BoC (cBoC, eBoC) due to the convergence of locally generated ACEs that interacted with the CE. Miron et al. [42] performed a Markov Chain analysis of surface drifter trajectories deployed throughout the GoM and showed the BoC has a relatively short “invariance time scale” compared to the Louisiana-Texas and Florida shelves and slopes, indicating somewhat limited retention capabilities and greater connectivity with the central Gulf compared to the other regions. Furthermore, dispersion studies of surface drifters in the BoC by Zavala-Sansón et al. [43–45] described two of the dominant circulation patterns observed in the BoC. Lower retention is observed when LC-ACE interacts with the BoC’s CE, leading to rapid northward transport primarily along the western margin of the Bay. In contrast, the absence of LC-ACEs in the BoC is associated with greater retention. Although these circulation patterns likely influence larval dispersal pathways and connectivity in the wGoM [14], the relationship between where larvae are found, their dispersal, and connectivity between different regions of the Gulf needs to be examined in further detail.

This study uses a high-resolution ocean circulation model to examine the dispersal pathways of virtual larvae (passive particles) from potential larval habitats of four species with contrasting life history strategies in the GoM’s deep-water region south of 25 ºN (within Mexico’s Exclusive Economic Zone; EEZ). First, we predicted potential habitats for the larvae for each species based on previously developed empirical relationships between larval density and environmental parameters. Dispersing particles from potential habitats, rather than randomly, provides a means for examining the dispersal pathways from areas likely to be occupied by the larvae of each species. Second, we examined the retention and dispersal pathways over 15 and 30 days (representative of larval stage duration) and for two periods with contrasting mesoscale circulation: (1) a high level of LC intrusion, absence of a recently detached LC-ACE, and no interaction between AE and CE in the BoC, and (2) limited LC intrusion, the presence of a recently detached LC-ACE in the central Gulf, and interaction between AE and CE within the BoC. As has been previously described, strong interactions (i.e. proximity) between mesoscale features within the BoC should lead to less retention, and a high level of intrusion of the LC into the GoM will transport virtual larvae from north of the Yucatan Peninsula through the Florida Straits to the Atlantic Ocean, compared to greater retention within the Gulf during limited LC intrusion. The results were compared to a null model in which particles were seeded throughout the BoC. Third, connectivity matrices were examined to evaluate the connectivity between regions of the GoM as a function of the location of potential habitats and the position of mesoscale features, including the LC. We posit that the location of species-specific potential habitats relative to mesoscale features and interactions will influence virtual larval dispersal and connectivity.

## Materials and methods

### Oceanographic conditions selected for particle tracking experiments

**Fig 1:**
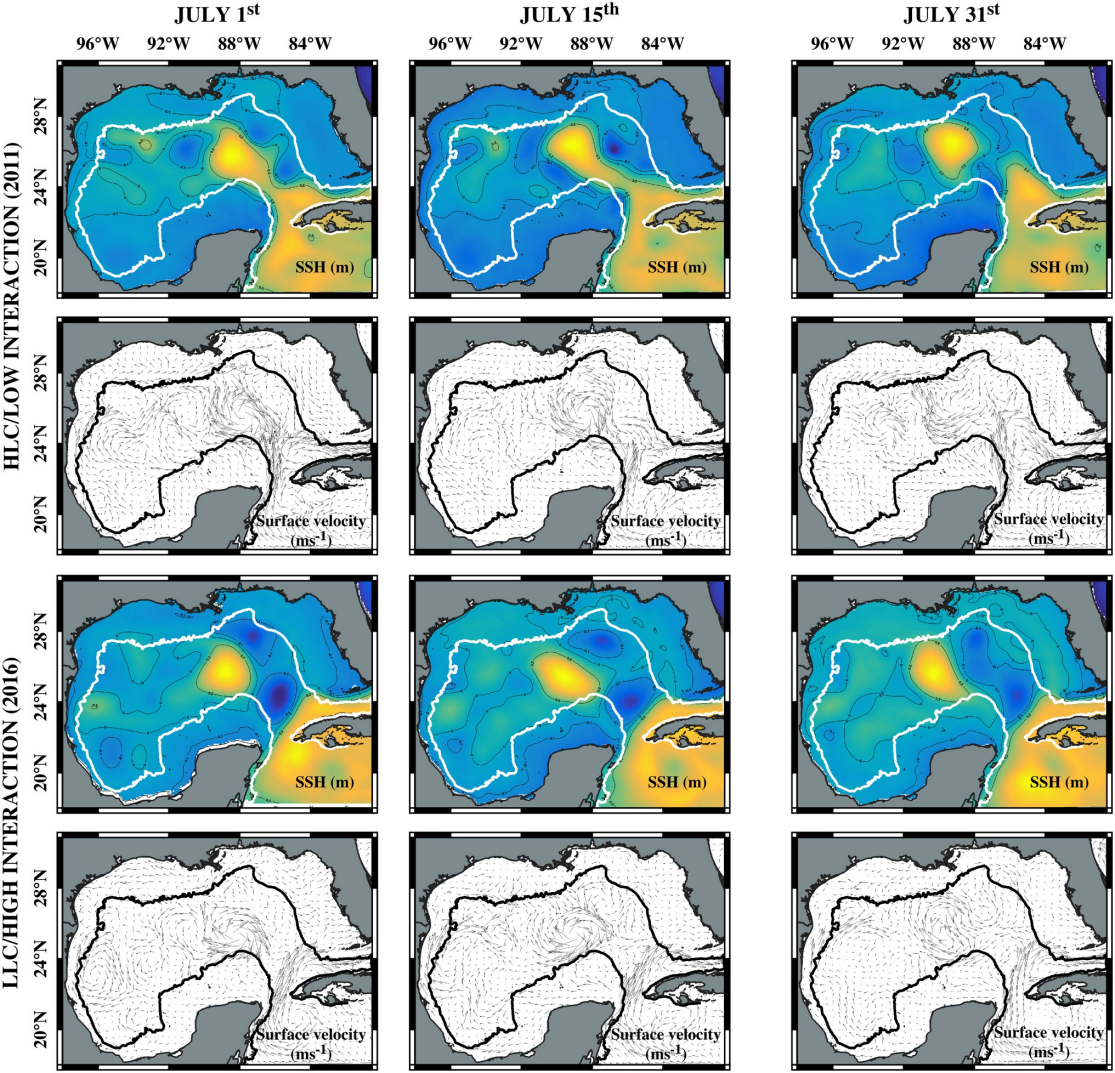
Surface circulation and mesoscale features in the GoM’s deep-water region during particle tracking experiments. HLC/low interaction (two upper rows): High Loop Current intrusion/low interaction between mesoscale features (2011). LLC/high interaction (two lower rows): low Loop Current intrusion/high interaction between mesoscale features (2016). July 1^st^ (col. 1), July 15^th^ (col. 2), and July 31^st^ (col. 3). Colored maps with contours (-0.1, 0, and 0.2 m) represent sea surface height (SSH, m). Vector maps indicate surface velocities (m s^-1^).

The study area for the dispersion experiments encompassed the GoM’s deep-water region (depths >1000 m; Fig 1). Two contrasting conditions during the summer season were selected for the experiments: (1) July 1^st^ to 30^th^ 2011, when there was a high LC intrusion and no recently detached LC-ACE in the central Gulf, the BoC’s CE was relatively small and weak, and there was no interaction between mesoscale features (hereafter referred to as HLC/low interaction), and (2) July 1^st^ to 30^th^ 2016, when the LC’s eddy Olympus (“huge” size category, https://www.horizonmarine.com/loop-current-eddies) had detached and traveled westward. There was interaction between the BoC’s CE and ACEs due to their proximity (hereafter referred to as LLC/high interaction). The July 1^st^ to 30^th^ period was selected for characterizing dispersal trajectories and connectivity based on the target species’ spawning seasons (they are year-around spawners or show summer spawning peaks; see Table 2 from [46]. Since the study aimed to characterize the differences in larval dispersion among contrasting conditions, only one example of each condition was used in the dispersion models. As described in previous studies, the circulation patterns we selected represent dominant circulation patterns in the GoM, particularly within the BoC [27, 30, 33]. Dispersal patterns would vary under differing circulation patterns.

### Prediction of potential larval habitat from hydrographical parameters

The seasonal variations in circulation patterns and environmental conditions modify larval habitat suitability, influencing species’ distribution, density and dispersal. Adults of neritic species mainly occupy habitats from the coastline to the continental shelf’s edge (200 m) [47, 48]. However, neritic species can be found among those mesopelagic species in the GoM’s epipelagic deep-water region (depths >1000 m) due to larval offshore transport toward oceanic waters [6, 36]. In contrast, adults of oceanic taxa can be classified as epipelagic (0–200 m), mesopelagic (200–1000 m), and bathypelagic (1000–4000 m) [48]. We modeled the potential larval habitat of *Notolychnus valdiviae* (lanternfishes; Myctophidae; mesopelagic), *Cubiceps pauciradiatus* (drift fishes; Nomeidae; epipelagic), *Bregmaceros atlanticus* (codlets; Bregmacerotidae; epi- and mesopelagic) and *Auxis* spp. (Scombridae; neritic and epipelagic). *Auxis* spp. groups larvae of *A*. *rochei rochei* and *A*. *thazard thazard*, known as bullet and frigate tuna, respectively. Both *Auxis* species were grouped since their larvae cannot be distinguished morphologically during the early life stages, and molecular identification is necessary [49, 50]. Nevertheless, both species share similar distribution, habitat, depth range and spawning periods in the GoM [47, 51, 52].

To predict each species’ potential habitat in the GoM’s Mexican EEZ (south of 26 ºN), a ten-year climatology spanning 2009 to 2018 and late spring and summer (April 1^st^ to July 31^st^) was constructed from daily remote sensing-derived variables (sea surface temperature (SST), sea surface height (SSH), surface chlorophyll-a concentration (chl *a*), and wind speed from the E.U. Copernicus Marine Service (https://data.marine.copernicus.eu/products) [53]. The 0 to 200 m mean salinity was obtained from the Hybrid Coordinate Ocean Model (HYCOM) reanalysis model outputs (https://tds.hycom.org/thredds/catalog.html) [54], and stratification was calculated according to Simpson et al.’s [55] equation with data obtained from HYCOM and is reported in Joules m^-3^; 0 corresponds to a well-mixed layer and the value increases with stratification.

The environmental variables used to predict larval potential habitats matched the hydrographic variables (including their spatial resolution) and the seasonality of the GAMs reported by Daudén-Bengoa et al. [46]. Briefly, in that study, GAMs were fitted to environmental data and larval density and distribution from 6 oceanographic cruises conducted between April and July (2011–2017). All stations were in depths >1000 m. Hence, potential habitat predictions refer exclusively to the larval stage. The environmental parameters were wind speed, chl *a*, SST, and SSH from remote sensing data products (E.U. Copernicus Marine Service Information). Wind speed was extracted from the ERA5 dataset downloaded from COPERNICUS. Mean salinity 0–200 m and stratification were calculated from CTD data, bottom depth from the ETOPO1 1 Arc-Minute Global Relief Mode, and tow coordinates were obtained from cruise records. Resulting GAMs for each species are reported in S1 Table. No permits are required for larval fish collections in Mexican waters.

Since the spatial resolution of the hydrographic variables of some of the environmental parameters used in the original GAMs and the data sources used to predict differed (for example, SST and SSH are available at 0.25º x 0.25º resolution and HYCOM outputs are at 0.08º x 0.08º), gridded explanatory variables were re-scaled to the coarser resolution (0.25º x 0.25º). The predict.gam function from the “mgcv” package [56] was used to predict the species’ density in potential habitats using the 10-year climatology in R [57].

**Fig 2:**
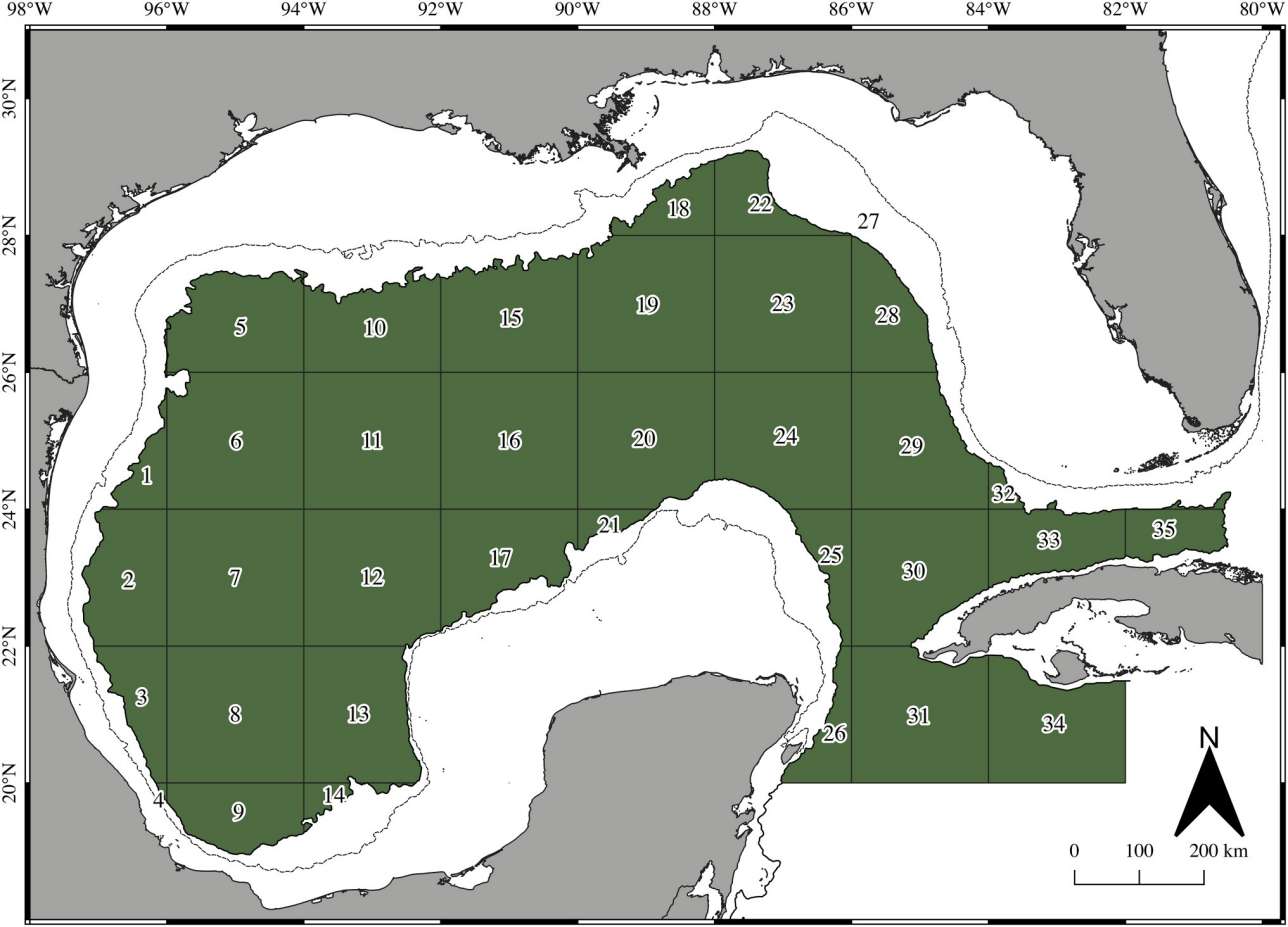
Quadrants used to examine particle dispersion in the GoM consisting of a 2 by 2-degree grid covering the deep-water region (depths >1000 m). Numbers indicate particle arrival quadrants. The red polygon represents the BoC’s null model. Dashed and continuous lines represent the 200, and 1000 m isobaths.

A subset of each species’s larval predicted potential habitat was used for particle seeding experiments to focus on the areas with the highest predicted larval density. In addition, larval densities were normalized to facilitate visualization of dispersal patterns and simplify the comparison between species. Normalized densities were obtained by scaling each species’ predicted densities (where 1 represents the maximum predicted density and 0 the absence of larvae). Particles were seeded only where the normalized predicted density (PD) was >0.4 (hereafter PD >0.4); this value was considered optimal because by using a value of 0.3, the subset area was small compared to the predicted habitat, and 0.5 yielded a very broad potential habitat (data not shown). Since the BoC has been described as a retentive region based on oceanographic modeling and statistical analysis of surface drifter data [42, 58], a null model encompassing the entire BoC’s deep water region (south of 22 ºN) was included in particle seeding experiments for comparison to species-specific potential habitats (Fig 2).

### Particle tracking experiments

The HYCOM + NCODA reanalysis circulation model was used for numerical simulations (https://www.hycom.org/data/gomb0pt04/gom-reanalysis) [54] which provides horizontal velocity components (*u* and *v*, eastward and northward respectively). As used in Compaire et al. [6], the velocity fields values in our study corresponded with the smallest spatial and temporal resolution available (0.04º x 0.04º, ~4 km horizontal resolution and 1 hour temporal resolution) to reduce simulation errors. Since neritic and mesopelagic larvae inhabit the upper water column during their development [59, 60], from the 40 depth levels that HYCOM + NCODA model provides, seeded particle’s dispersal pathways were tracked using five velocity (*u*, *v*) layers between 0 and 200 m (0, 50, 100, 150 and 200 m). Only horizontal velocities (*u*, *v*) were considered since there is little information on the larvae’s vertical distribution and swimming patterns in the water column; seeding particles at 5 depths simulated dispersal between 0 and 200m. Additionally, passive particle advection was calculated using a fourth-order Runge-Kutta algorithm [6]. Vertical velocities in open ocean systems are much smaller than horizontal velocities [40, 61, 62]. For example, Zhong and Bracco [62] calculated sub-mesoscale vertical velocities for the open ocean of the GoM. They estimate maximum instantaneous vertical velocities at the margins of mesoscale eddies and fronts of around 10 m day^-1^ and 7 day averages of 2.5 m day^-1^ at 5 m depth, and up to 25 m day^-1^ and 5 m day^-1^, respectively, at 100 m depth. In contrast, upper layer circulation in the open waters of the GoM (excluding the Loop Current) ranges roughly from 0.1 to 0.3 m sec^-1^ (8–24 km d^-1^). Hence, we assume vertical velocities are negligible relative to horizontal velocities in terms of their influence on larval fish dispersal over the large spatial scales examined in this study.

The number of randomly seeded particles at t = 0 days (July 1^st^ 2011 and 2016) was proportional to the area of each potential habitat subset, where the smallest area *B*. *atlanticus* 1 (1076 km^2^) was assigned 2500 particles (500 at each depth; Table 1). The largest potential habitat was over 12 times larger (*N*. *valdiviae*) and seeded with 31,565 particles. For *B*. *atlanticus*, two habitat subsets were obtained from PD >0.4. To calculate particle transport throughout the GoM, 35 quadrants were generated by gridding the entire GoM’s deep-water region into a 2 by 2-degree latitude and longitude matrix (Fig 2).

**Table 1:** Area of potential habitats subset with each species’ predicted densities >0.4 and number of particles deployed at the 5 depth layers (z: 0, 50, 10, 150, and 200 m). Areas were scaled to that of the smallest potential habitat (*Bregmaceros atlanticus* 1) for comparative purposes.

Species	Potential habitat seeding area (km^2^)	Area relative to smallest habitat subset	Number of seeded particles at t = 0 days
***Bregmaceros atlanticus*** (1)	1076	1	2500
***Bregmaceros atlanticus*** (2)	2530	2.43	6080
***Auxis* spp**.	2815	2.62	6545
***Cubiceps pauciradiatus***	2943	2.80	6995
***Notolichnus valdiviae***	13462	12.63	31565
**BoC** (null model)	11999	11.23	28065

Particles were advected forward for 30 days, based on the average development time for neritic and mesopelagic larvae [63, 64]. Additionally, a midpoint (t = 15 ays) was also examined to aid the interpretation of the particle’s dispersion evolution through time. Given that the age of the larvae at the time of capture was unknown, a 15 day advection period also informed dispersal potential. The same dispersal periods (15 and 30 days) were applied to all species, and the null model allowed the evaluation of particle dispersion without the confounding effect of varying larval stage duration.

However, we acknowledge that this ignores the fact that greater dispersal distances can be achieved in species with longer larval stage duration, which has been shown to increase connectivity [9]. In the passive particle dispersion models, the number of particles present at each depth (*z*) in a quadrant (*j*) at a given time (t = {0:1/24:30}; 31 days with hourly resolution) was counted (*N*_*j*_ (*t*), with *j* = [1,2, …, 35] and then averaged over the five depths to obtain the percentage (%) of particles that arrived at each quadrant.

To determine retention and particle dispersal, the percentage of particles (*P*) that (1) remained within each species’ habitat subset (PD >0.4), (2) those that were dispersed within the deep-water region (*N*_*i*_; i.e., particles that stayed at locations with depths equal or greater to 1000 m), and (3) particles that were dispersed to depths shallower than the 200 m isobath (*N*_*t*_, i.e., transported onto the shelves) was calculated. Specific habitats, such as reefs, the seabed, or shallow water habitats, were not considered as our interest was in broad transport patterns and these species recruit to the pelagic habitat. Additionally, *N*_*t*_ and *N*_*i*_ were used to calculate the particles that reached the slope between the 200 and 1000 m isobath since some species can be found over the slope, which presumably provides adequate habitat (e.g. *Auxis* spp. and *B*. *atlanticus*).

Particle spatial distribution maps (Figs 4 and 5) were created to help visualize the percentage of particles retained and the dispersion pathways from the BoC at t = 0 days through t = 15 days and t = 30 days. The percentage of particles arriving at each quadrant from each habitat subset was calculated for the end of the larval dispersal period.

## Results

### Species-specific potential habitats

**Fig 3:**
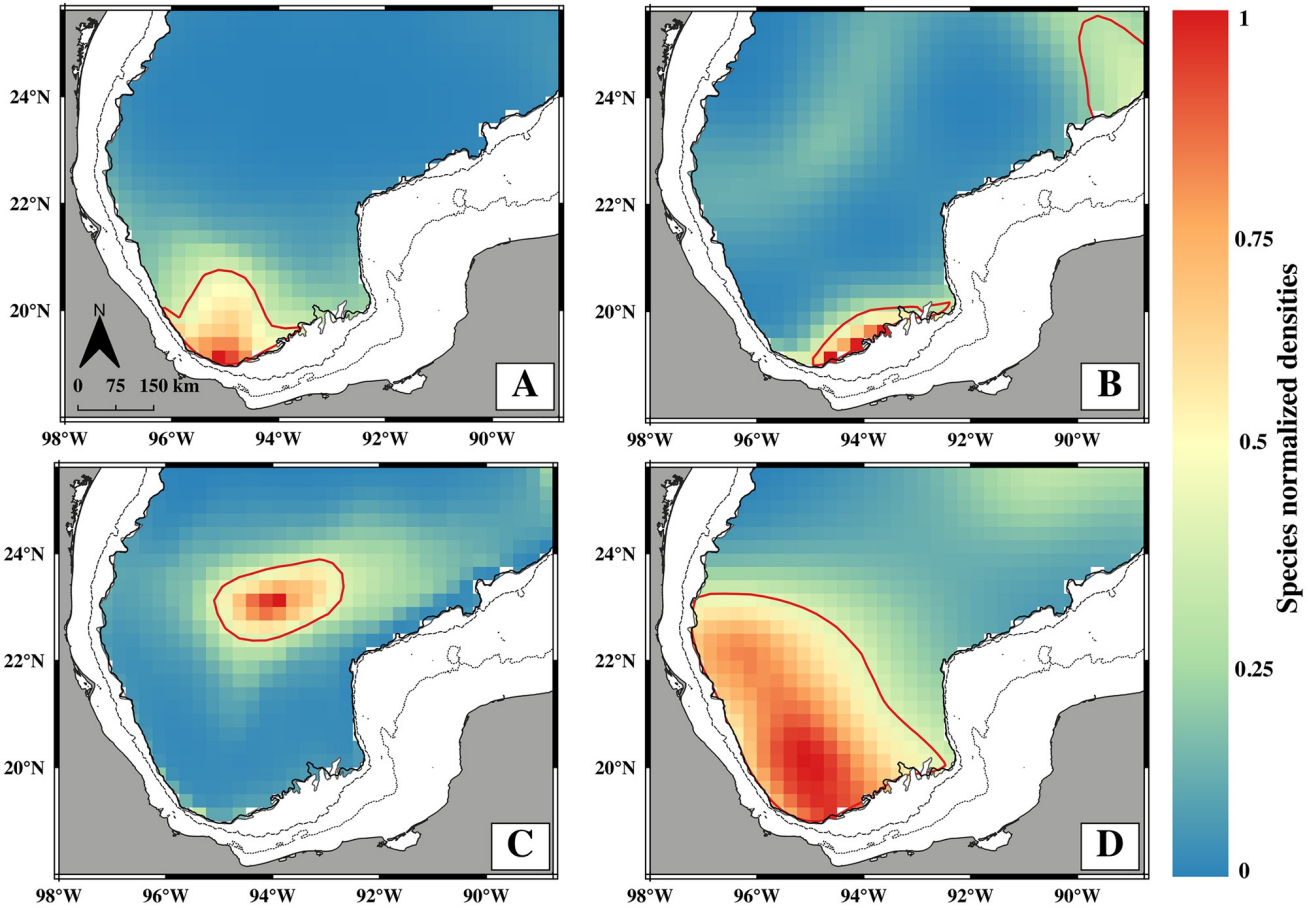
Species-specific larval potential habitats are expressed as normalized larval densities (maximum predicted density = 1). A: *Auxis* spp., B: *Bregmaceros atlanticus*, C: *Cubiceps pauciradiatus* and D: *Notolychnus valdiviae*. Red bold lines indicate habitat subsets (predicted densities >0.4) used in particle seeding experiments. Two potential habitat areas with PD >0.4 were predicted for *B*. *atlanticus* in the deep-water region. Dotted, dashed, and continuous lines represent the 40, 200, and 1000 m isobaths, respectively.

Each species’ full potential larval habitat had a distinct spatial distribution, reflecting the differing relationships between environmental conditions and larval density found with the GAMs (Fig 3; Dauden-Bengoa et al. [46]). Potential habitat subsets (normalized densities >0.4) for *Auxis* spp. and one of the two subsets for *B*. *atlanticus* were limited to the sBoC, and the greatest relative density of larvae was predicted to be found close to the continental slope. *N*. *valdiviae* had a more extensive potential habitat subset encompassing most of the BoC. In comparison, the subset for *C*. *pauciradiatus* was located strictly north of the BoC. The largest potential habitat subset corresponded to *N*. *valdiviae* with an extension of 13,462 km^2^, while the smallest was for *B*. *atlanticus* in the BoC with 1,076 km^2^ (Table 1). The second subset was predicted for *B*. *atlanticus* north of the Yucatan Shelf which was 1.5 times larger than the one within the sBoC.

### Simulation of larval transport

**Fig 4:**
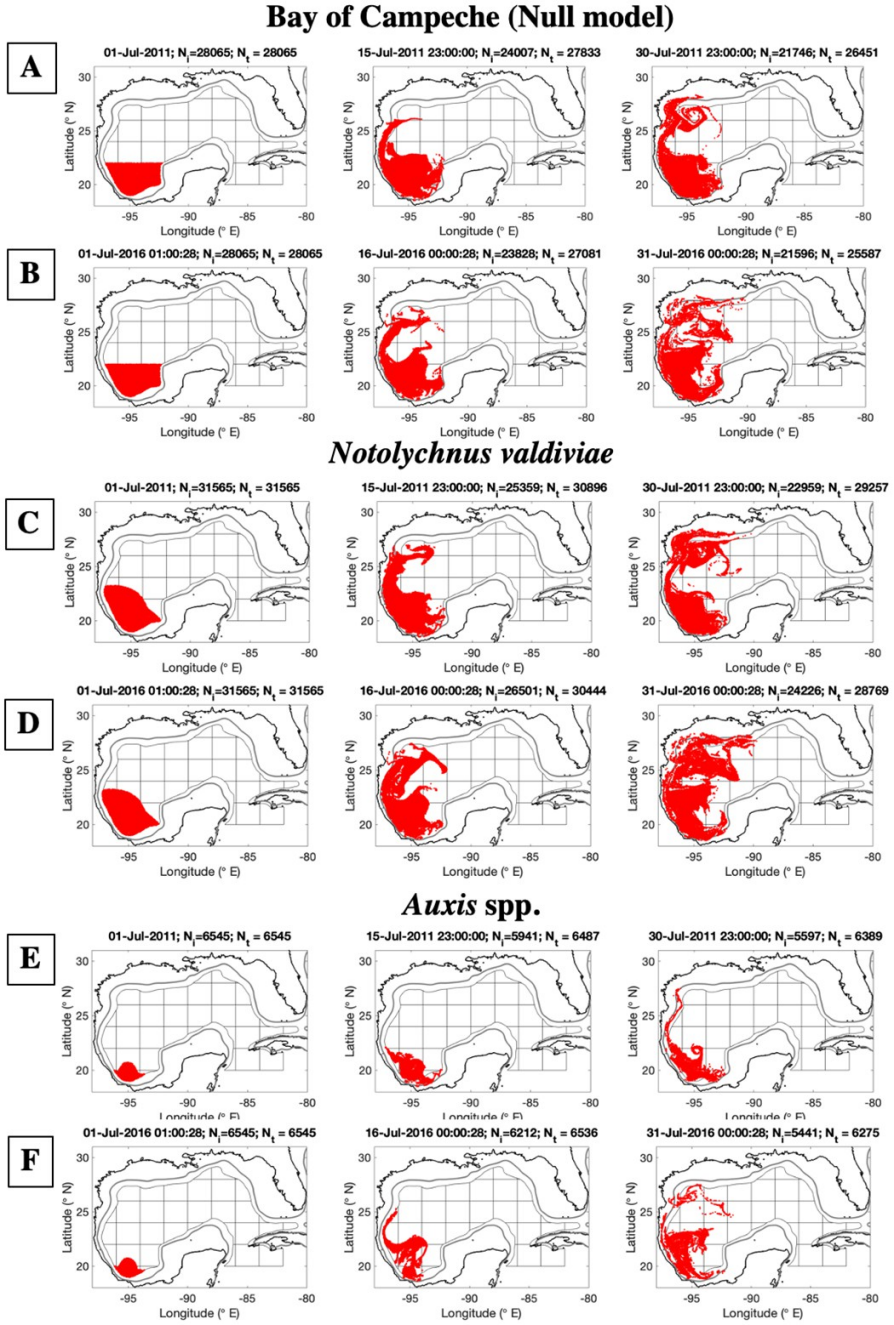
Results of particle dispersion experiments for the BoC’s null model (A, B), *N*. *valdiviae* (C, D) and *Auxis spp*. (E, F). First column: seeded particles at t = 0 days, second column: t = 15 days, third column: t = 30 days. HLC/low interaction (A, C, E): high Loop Current intrusion/low interaction between mesoscale features (2011). LLC/high interaction (B, D, F): low level of intrusion of the Loop Current/high interaction between mesoscale features (2016). Seeded particles (red dots) were released at five depths (z = 0, 50, 100, 150, and 200 m). *N*_*i*_: Number of particles retained at depths >1000 m. *N*_*t*_: Number of particles retained at depths >200 m and deeper (including the deep-water region). Smoothed 200 m isobath (gray continuous line), and smoothed 1000 m isobath (black line surrounding the 2 by 2-degree grid).

The dispersion of the seeded particles in the null model was limited to the wGoM (west of 92 ºW). Under HLC/low interaction (2011), most particles were transported toward the north along the western continental slope during the first 15 days; after 30 days, particles reached the northwestern shelf and a few were dispersed to the central Gulf due to the presence of a non-LC ACE in the nwGoM (Fig 4A). By contrast, under LLC/high interaction (2016), the northward transport after 15 days extended throughout the deep-water region (Fig 4B), and some particles were also dispersed to the shelf (<200 m). For *N*. *valdiviae*, the dispersal pattern was similar to that observed for the BoC’s null model (Fig 4C and 4D), reflecting the large overlap in the spatial distribution of its potential habitat subset and the spatial extent of the Bay. However, more particles reached the north-central GoM (28 ºN, 90 ºW) after 15 and 30 days from *N*. *valdiviae’s* habitat subset than from the BoC, which is due to its extent north of 22 ºN.

**Fig 5:**
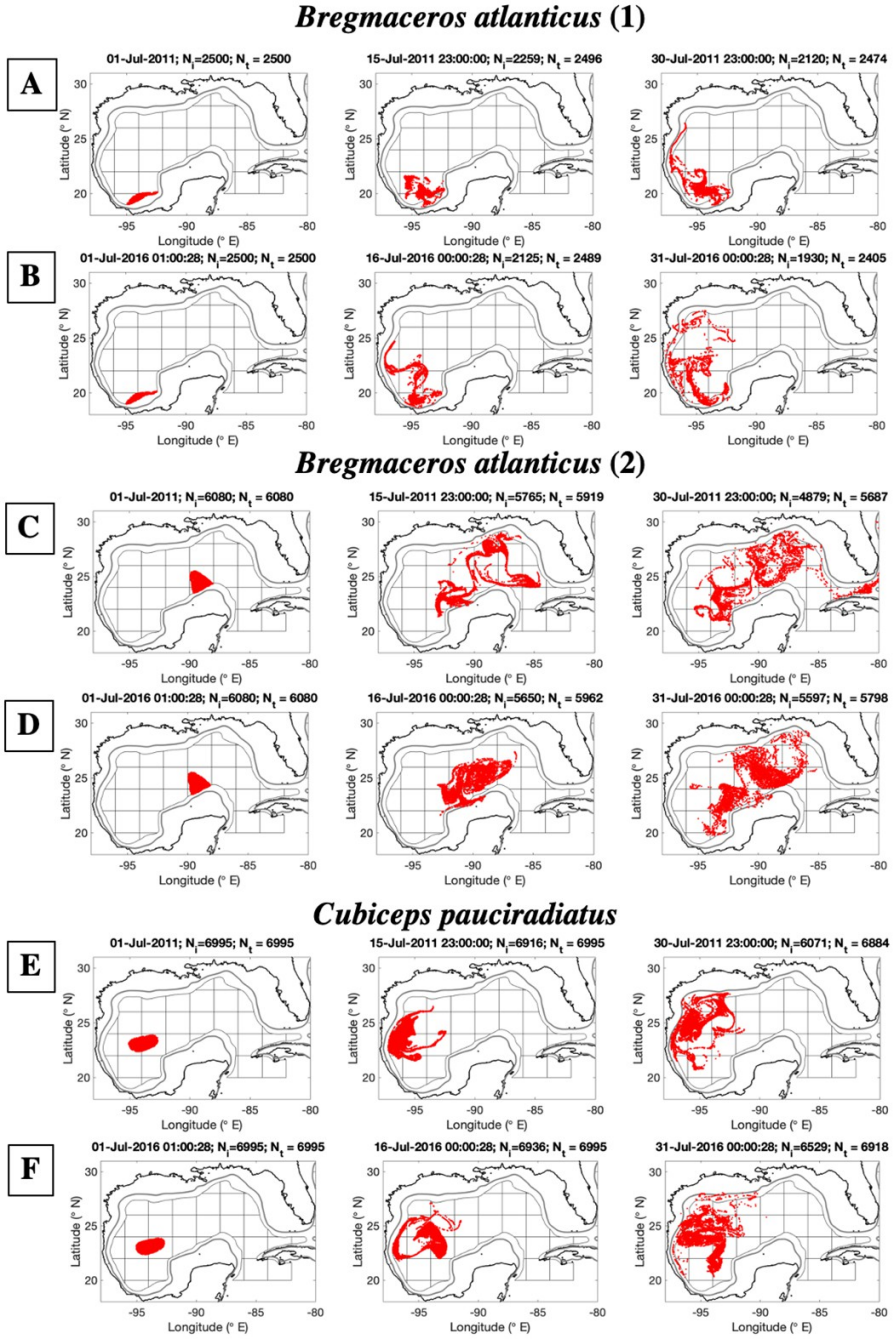
Results of particle dispersion experiments for *Bregmaceros atlanticus* 1 (A, B), *B*. *atlanticus* 2 (C, D) and *Cubiceps pauciradiatus* (E, F) over gridded regions. See Fig 4 for the caption’s description.

In comparison, particles seeded for *Auxis* spp. (Fig 4E and 4F) and 4*B*. *atlanticus* in the sBoC (Figs 4B and 5A) were transported northward along the western continental slope, but their dispersal was less extensive than for *N*. *valdiviae’s* and the BoC’s null model. For *Auxis* spp., after 15 days of dispersal under HLC/low interaction, the particles remained within the BoC, with very few particles transported to the upper slope (Fig 4E). However, after 30 days, some particles reached the nwGoM (including the shelf) due to northward along-slope transport. In comparison, during LLC/high interaction (Fig 4F), at 15 days post-release particles had traveled northward and reached 25 ºN along the GoM’s western slope, and after 30 days, particles reached the nwGoM (including the shelf). Additionally, particles that reached the nwGoM were dispersed eastward by anticyclonic circulation in the western Gulf. The distribution of the dispersed particles from the sBoC subset of *B*. *atlanticus* (1), was similar to that of *Auxis* spp., which is to be expected based on the similarity in the spatial distribution of their potential habitat subsets.

The dispersal of particles from the *B*. *atlanticus* (2) habitat subset located north of the YS (Fig 5C and 5D) indicated that after 15 days there was more widespread particle advection under HLC/low interaction compared with LLC/high interaction. When the LC extended far into the Gulf, particles reached the nGoM’s shelf near the Mississippi Delta as well as the eastern BoC (Fig 5C). By 30 days post-release, particles were transported through the Florida Straits and particles had dispersed toward shallower waters beyond the 1000 m isobath (14%) and onto the continental shelf (7%). In contrast, the low LC intrusion led to greater retention of particles close to the YS near the potential habitat, with some advection to the sGoM as far as the southern potential habitat (e.g., *B*. *atlanticus* 1). The low LC intrusion also led to more particles remaining inside the deep-water region (HLC/low interaction: 79%; LLC/high interaction: 92%).

The overall dispersion from *C*. *pauciradiatus’* potential habitat subset also differed between circulation patterns. Under HLC/low interaction, particles traveled westward after 15 days (Fig 5E), while under LLC/high interaction (Fig 5F), particles remained close to the potential habitat subset due to retention by an anticyclonic feature in the northern BoC. Under HLC/low interaction, particles were transported away from the potential habitat toward the Texas shelf, with some particles reaching the continental slope. After 30 days of dispersion, under LLC/high interaction, more particles were retained or advected back to the habitat subset.

The percentage of particles retained within potential habitat subsets varied between species and between circulation patterns; retention was higher for *B*. *atlanticus* (2) (north of the Yucatan shelf) and *C*. *pauciradiatus* when there was a low level of LC intrusion and high interaction between mesoscale features (Table 2). In comparison, retention within the potential habitat subsets was higher for *N*. *valdiviae* and the BoC’s null model under HLC/low interaction. None of the seeded particles were retained for *Bregmaceros atlanticus* (1). Retention of particles in the deep water region was high for all species (>72%), but there was also transport to the upper slope (4–20%) and onto the shelf (1–9% of particles) for both scenarios. The BoC’s null model dispersal pattern indicated retention within the Bay was greater under HLC/low interaction, which is consistent with the descriptions of Zavala-Sansón et al. [43–45]. Specifically, under LLC/high interaction, more than 50% of particles were dispersed outside the BoC after 30 days.

**Table 2:** Percentage of seeded particles that were retained at t = 30 days within the potential habitat subset, in the GoM’s deep water region (>1000 m), or transported to the upper slope (200 to 1000 m) or continental shelf (<200 m). Bold values indicate the largest value for each category. HLC/LI: high Loop Current intrusion/low interaction between mesoscale features (2011). LLC/LI: low intrusion of the Loop Current/high interaction between mesoscale features (2016).

Species	Retained in potential habitat subset (%)		Retained in the deep-water region (%)		Dispersed to upper slope (%)		Dispersed to continental shelf (%)	
	HLC/LI	LLC/HI	HLC/LI	LLC/HI	HLC/LI	LLC/HI	HLC/LI	LLC/HI
*Auxis* spp.	13	**18**	**87**	84	11	11	2	**5**
*Bregmaceros atlanticus* (1)	0	0	**86**	76	13	**20**	1	**4**
*Bregamceros atlanticus* (2)	55	**73**	79	**92**	**14**	4	**7**	4
*Cubiceps pauciradiatus*	15	**52**	87	**93**	**11**	6	**2**	1
*Notolychnus valdiviae*	**66**	47	72	**77**	**20**	14	8	**9**
BoC (null model)	**40**	30	77	77	**17**	14	6	**9**

For *B*. *atlanticus* (2) habitat subset north of YS and *C*. *pauciradiatus*, no particles entered the BoC after 15 days under LLC/high interaction. However, after 30 days, 9% of the released particles for *B*. *atlanticus* (2) reached the BoC under HLC/low interaction (vs. 6% under LLC/high interaction). For *C*. *pauciradiatus*, 12% reached the BoC under LLC/high interaction (and 5% under HLC/low interaction) after 30 days. This suggests there is potential for larval transport from the north of the YS and the central GoM to the BoC (Table 3).

**Table 3:** Percentage of particles retained in the Bay of Campeche (BoC) at t = 0 days, at t = 15 days and t = 30 days. HLC/LI: high Loop Current intrusion/low interaction between mesoscale features (2011). LLC/HI: low intrusion of the Loop Current/high interaction between mesoscale features (2016). Bold values indicate the highest value for each comparison.

	Particles retained in the Bay of Campeche					
Species	t = 0 days (%)		t = 15 days (%)		t = 30 days (%)	
	HLC/LI	LLC/HI	HLC/LI	LLC/HI	HLCLI	LLC/HI
*Auxis* spp.	100	100	**100**	73	**94**	50
*Bregmaceros atlanticus* (1)	100	100	**100**	64	**87**	48
*Bregmaceros atlanticus* (2)	0	0	**2**	1	**9**	6
*Cubiceps pauciradiatus*	0	0	**7**	5	5	**12**
*Notolychnus valdiviae*	72	72	**68**	57	**61**	33
BoC (null model)	100	100	**84**	74	**73**	44

**Fig 6:**
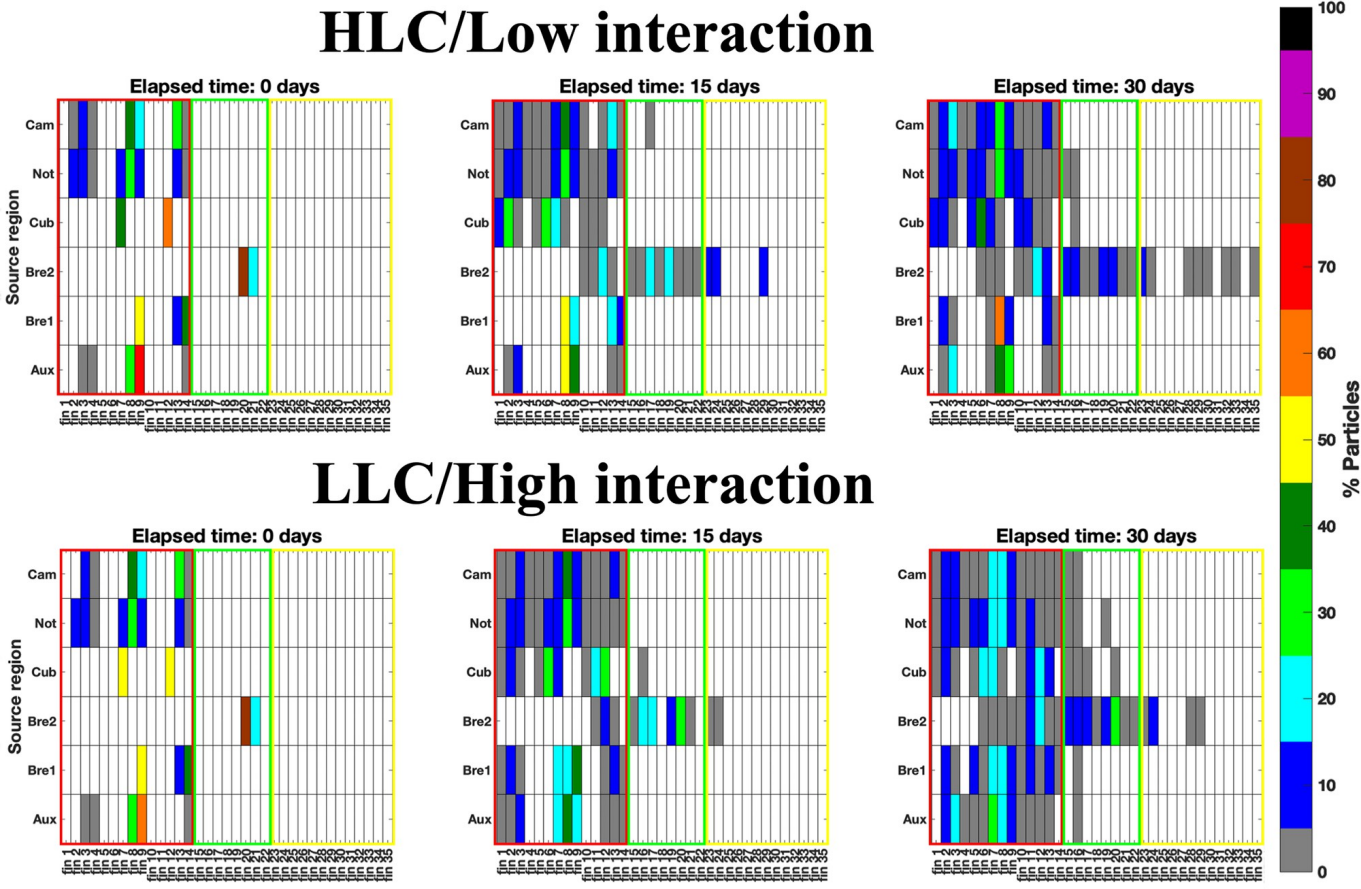
Species’ particle distribution matrix after t = 0 days, t = 15 days and t = 30 days under HLC/low interaction between mesoscale features (top) and LLC/high interaction between mesoscale features (bottom). Rows from top to bottom: BoC (Bay of Campeche), Not (*N*. *valdiviae*), Cub (*C*. *pauciradiatus*), Bre 1 (*B*. *atlanticus* (1) in the sBoC) and Bre 2 (*B*. *atlanticus* (2) north of YS) and Aux (*Auxis* spp.). Columns: quadrant numbers from Fig 2. Red box: western GoM (-98 to -92 ºW); green: central GoM (-92 to -88 ºW); yellow: eastern GoM (-88 to -80 ºW).

The differences in size and location of the species’ potential habitat subset led to differences in the connectivity among GoM’s regions after 30 days (Fig 6). The connectivity matrices indicated that the particle dispersion and connectivity were largely limited to the BoC and the wGoM, except for the potential habitat subset of *B*. *atlanticus* (2) located north of YS. This species had the greatest connectivity, with particles dispersed to the sBoC, the wGoM and the Florida Straits.

For the rest of the potential habitat subsets, dispersion was mainly limited to the wGoM. *C*. *pauciradiatus’* connectivity with the north-central GoM was mainly under LLC/high interaction. The greatest percentages of particles remained in the wGoM, and few reached the southernmost BoC. For the BoC null model, dispersal was greater under LLC/high interaction as expected, indicating greater connectivity when compared with HLC/low interaction. While *N*. *valdiviae* and the BoC’s null model presented dispersal limited to the wGoM after 30 days, for *Auxis* spp. and *B*. *atlanticus* (1) there was connectivity between sBoC and the deep waters of the nwGoM during LLC/high interaction. In contrast, under HLC/low interaction, particle dispersal was limited to the GoM’s western slope, and the connectivity level with other Gulf areas was more limited.

## Discussion

Our results highlight the importance of using a comparative approach centered on species with different potential habitats coupled with Lagrangian dispersion models to understand the dispersal and connectivity of marine fish larvae. The size and location of predicted habitat subsets played a crucial role in dispersal patterns and connectivity between regions of the GoM’s deep-water region. Overall, transport toward the northern GoM along the shelf was observed from predicted habitat subsets in the sBoC. Larvae from habitat subsets that occupied most (*N*. *valdiviae*) or all of the BoC (null model) were dispersed more homogenously toward the north. By contrast, larvae from more spatially limited habitats within the BoC showed greater retention. Additionally, comparing the dispersal and connectivity between distinct circulation patterns (HLC/low interaction during 2011 and LLC/high interaction in 2016) also indicated a strong influence in the dispersion paths from each predicted habitat subset, leading to distinct levels of connectivity and retention within the GoM.

The prevalence and seasonality of (1) the occurrence or lack of interaction between AE and CE in the BoC, and (2) the high or limited LC intrusion into the GoM will logically influence larval dispersal patterns. Olvera-Prado et al. [40] documented LCE separation events between January 1997 and December 2015 (see Table 2). Of 34 LCE formation events over 18 years, 17 (50%) reached the BoC. Assuming 1/3 of their mean lifetime (311 days) was within the BoC, LCEs would be in the BoC about 28% of the time (1762/6205 days). Guerrero et al. [31] also estimated that LCEs interact with the cyclonic eddy of the BoC about 30% of the time. Therefore, the lack of interaction between LC-ACEs and the semi-permanent cyclonic eddy should be more prevalent, and northward larval dispersion along the western margin of the BoC would be less frequent than retention within the Bay. Both circulation patterns are nevertheless representative, as has been shown through the analysis of surface drifters [33, 65]. We found a high level of LC intrusion leads to particle dispersal to the northeastern GoM and through the Florida Straits. LCE detachment has been documented throughout the year but is statistically higher in August and September and possibly February and March [66, 67]. Larval transport from the north of the Yucatan Shelf through the Florida Straits is possible throughout the year, but more likely during the summer. Nevertheless, further studies are warranted to confirm those expectations and characterize the influence of other circulation features on larval fish dispersal in the GoM’s deep-water region.

### Species-specific potential habitat predictions

Reports of the larval distribution of the four taxa indicate that the potential habitat predictions adequately represent the habitats in which they are found. The predicted potential habitat of *Auxis* spp. was in the deep waters of the sBoC near the continental slope. This agrees with previous studies in the area during summer [68, 69]. *Auxis* spp. larvae have also been caught beyond the slope in other GoM’s regions, such as in the nGoM off the western Florida Shelf [70], and in the region of influence of the LC [28]. The presence of *Auxis* spp. larvae in the deep waters of the BoC could be due to (1) adult spawning in oceanic waters despite being a mainly neritic species [51, 71] and/or (2) seasonal offshore cross-shelf transport in the south-eastern BoC [38] coupled with high river runoff from the Grijalva-Usumacinta during periods of along-shelf convergence in the south-western BoC [39]. However, little is known about the distribution of larvae and adults of *Auxis* spp. Pruzinsky et al. [72] noted that *Auxis thazard* (among other neritic scombrids) in the nGoM preferred areas with greater chl *a* concentration, closer to the slope, and with lower salinities. This is consistent with the presence of larvae in the sGoM, where there is seasonally high freshwater inflow and offshore transport [38].

The distribution of *N*. *valdiviae*’s predicted potential habitat agrees with its previously reported distribution [73] since it is a cosmopolitan mesopelagic species found throughout the GoM’s deep-water region [63, 73, 74]. The highest larval densities were predicted for the BoC, which also agrees with previous studies for this and other mesopelagic species [69, 74, 75]. The BoC has a higher nutrient concentration near the surface and higher zooplankton biomass than the central GoM [76] and is considered more productive [77, 78]. Hence, the higher densities are attributed to the higher productivity in the BoC due to a shallowing of the pycnocline in the central BoC [32], wind-driven upwellings [30] and river runoff from the Grijalva-Usumacinta river in the southeastern BoC [39].

In contrast, *B*. *atlanticus* has been described as both a neritic and mesopelagic species that mainly occupies the continental shelf’s slope in the sGoM [69, 79, 80], southeastern BoC [81] and eGoM [82, 83], which agrees with the potential habitat predictions. The potential habitat of *B*. *atlanticus* off the northern YS could be due to larval offshore transport toward deep waters caused by the easterly winds driving westward circulation over the shelf throughout the year [84, 85]. Some studies have reported the presence of *B*. *atlanticus* larvae over the western Florida Shelf [82, 83] and of other *Bregmaceros* species in the sGoM’s shelf [80, 86]. However, little is known about *B*. *atlanticus* distribution in the GoM’s deep-water region and further studies are required.

The prediction of *C*. *pauciradiatus’* potential habitat spanning the deep waters of the northern BoC and the central GoM matches descriptions of a broad larval distribution in several GoM studies [87–89]. The predicted distribution is also consistent with their adult mesopelagic distribution [47, 90, 91].

The agreement between potential habitat predictions and the known distribution suggests potential habitat subsets are likely suitable for their growth and development and potential recruitment to adult populations. While using a 10-year climatology does not allow for the characterization of each species’ distribution over shorter time scales, grouping biological data from several cruises within a season in the GAMs [46] allows for a broader view of the overall species distribution, and more robust predictions of the species potential habitats. Potential habitats will logically differ between years due to spatial and temporal variability of environmental drivers.

### Simulation of larval transport

The spatial distribution of the potential habitats PD > 0.4 strongly influenced the dispersion of virtual larvae in the GoM’s deep-water region. Additionally, the location of mesoscale features and their interaction also controlled the retention and dispersion of the virtual larvae. Importantly, in natural populations, larvae can be dispersed toward suitable conditions that favor growth and survival or to unsuitable or disturbed areas where growth is limited and mortality is higher. However, over time, the spatial and temporal variability in dispersal patterns contributes to maintaining connectivity between areas with suitable conditions, which is key to larval development, survival, and ultimately recruitment success [9, 92, 93].

Under a circulation pattern with HLC/low interaction, there was a northward transport from the BoC along the western slope, along with the western boundary current linked to wind-driven circulation in the western GoM present from April to August, as previously reported by Zavala-Hidalgo et al. [30] and Guerrero et al. [31]. This northward transport was also observed in the western BoC’s continental shelf based on the analysis of surface drifters by Dubranna et al. [27] and by Pérez-Brunius et al. [33]. In this study, the absence of LC-ACEs under the HLC/low interaction condition led to the dispersion of the virtual larvae to the slope in the north wGoM (close to 26 ºN). This is also observed in a 10-year simulation of monthly mean surface currents of the overall northward circulation in the wGoM’s shelves due to wind stress and the local currents described by Zavala-Hidalgo et al. [30, 65]. The overall dispersion toward the nwGoM’s slope may positively influence larvae of the neritic species *Auxis* spp. In contrast, the transport of mesopelagic species (*C*. *pauciradiatus*, *N*. *valdiviae*) to the continental shelf may negatively affect survival if conditions are not conducive to recruitment.

Under LLC/high interaction, there was also northward transport along the western slope. However, strong advection toward the deep-water region was observed due to the slope’s interaction with anticyclonic circulation. This advection was also reported by Compaire et al. [6] based on the analysis of chl *a* plumes in surface waters from the shelf near the Perdido region (close to 26 ºN), and was accompanied by the transport of fish larvae of coastal and neritic species to the deep-water region. In addition, a northward transport from the central BoC was also observed under LLC/high interaction, which was previously described by Pérez-Brunius et al. [33] and Hamilton et al. [94], and was related to the intrusion of anticyclonic circulation into the BoC that interacted with semi-permanent CE.

The LC’s high level of intrusion increased the connectivity along the nGoM and between the wGoM’s and sGoM and advected particles from the central Gulf through the Florida Straits to the Atlantic Ocean. This has been observed in previous studies (e.g., [95, 96]). In comparison, the limited intrusion of the LC into the GoM seems to limit the dispersion of particles to the Atlantic. Therefore, variation in the intrusion level of the LC might disperse or retain larvae closer to suitable development and survival conditions in which they were caught.

### Retention, dispersion, and connectivity of the BoC with the nGoM

The species with potential habitat subsets in the southernmost BoC (*Auxis* spp. and *B*. *atlanticus*) had about ~50% more particles within the Bay after 30 days compared with *N*. *valdiviae* and the BoC’s null model, for which particles were seeded throughout the BoC’s deep water region. This indicates greater retention in areas limited to the sBoC. The greater retention after 30 days in the BoC under HLC/low interaction for all four species (> 60%) agrees with the circulation described by Perez-Brunius et al. [33]. Zavala-Sansón et al. [43–45] results indicate limited dispersion when ACEs are absent from the southern GoM and the “partial” retentive nature of the BoC inferred by Miron et al. [42]. Under LLC/high interaction, our results are also consistent with those studies since the interaction of anticyclonic features with the BoC increased the dispersion toward the north (high interaction: <50% retention after 30 days). The interaction between mesoscale features from the central Gulf and those in the BoC tends to increase larval dispersion and connectivity with the nGoM.

In this study, a high intrusion of LC transported virtual larvae from *C*. *pauciradiatus* potential habitat subset to the wGoM’s slope after 15 days due to westward circulation and low interaction with the BoC. Olvera-Prado et al. [97] reported a westward circulation by calculating the mean circulation and transport in the nGoM under the influence of a high intrusion level of the LC. Additionally, 13% of particles were dispersed further into neritic waters after 30 days, likely leading to the loss of those larvae since they are not found over shelf waters.

*C*. *pauciradiatus* is a mesopelagic species whose adult habitat preference is mainly near oceanic jets, such as areas where mesoscale features interact [87]. These are areas of higher productivity [98], in contrast with the more oligotrophic areas of the deep waters of the GoM [78, 99]. Under LLC/high interaction, the interaction of anticyclonic features from the nGoM near the 94 ºW with the CE in the BoC increased the retention near *C*. *pauciradiatus* potential habitat PD >0.4, within the BoC and in the deep-water region. Therefore, the greater percentage of particles in mesopelagic waters and within the potential habitat PD >0.4 under LLC/high interaction may support greater survival and recruitment of *C*. *pauciradiatus* larvae.

### Retention, dispersion and connectivity in the LC region

The variation in the intrusion level of the LC played a key role in the particle dispersion from *B*. *atlanticus* (2) potential habitat subset north of the YS. A higher LC’s level of intrusion dispersed more particles toward the wGoM and through the Florida Straits, suggesting the loss of some *B*. *atlanticus* larvae from the Gulf ecosystem to the Atlantic. Clancey [100] showed a high density of post-larval, juvenile and adult specimens of this species in the Florida Current throughout the year. Given that *B*. *atlanticus* is a cosmopolitan species distributed throughout the central Atlantic [47, 89], the intrusion of the LC may link populations within the GoM with those of the Atlantic if conditions in the Florida Current favor growth, survival and recruitment. The assessment of the implications of LC-driven connectivity of larval fish populations would have to be examined using chemical or genetic markers to examine population structure.

Lower LC intrusion showed greater retention near the predicted habitat of *B*. *atlanticus* (2) north of the YS, with no dispersal outside the GoM, but greater connectivity towards the sGoM. This may indicate a higher probability of survival due to the presence of particles (virtual larvae) in areas with more favorable conditions and higher productivity, such as the sBoC [37, 38]. Similar connectivity patterns have been described by Johnson et al. [5] in dispersion models applied to red snapper larvae (*Lutjanus campechanus*), where particles were dispersed from the YS toward the southern reaches of the BoC. A similar circulation pattern was observed by Sanvicente-Añorve et al. [14]; who observed particles tracked for 35 days traveled from the northern slope of the YS to the sBoC. The circulation pattern in these results and ours might indicate a connecting route between the northern YS and the sBoC for larval fishes.

These experiments may present several limitations, such as not incorporating vertical larval migration, which was addressed by modeling particle dispersion in several depths where vertical migration occurs, or horizontal larval transport, considering the contrast between the GoM’s average surface velocities and the limited larval swimming capacity. In addition, we did not include variations in the period of dispersion (e.g., larval development time) for each species due to limited information regarding their life history. On the other hand, these factors were not included in the models to isolate the effect of the size and distribution of the species’ predicted potential habitat and distinguish its implications for larval dispersion and connectivity. Biological factors, such as vertical migration, food availability, growth, and predation rates, contribute to recruitment success or failure. Quantifying and integrating these processes into individual-based Lagrangian particle dispersal models is challenging but necessary to improving the understanding of connectivity and recruitment to juvenile nursery habitats [13]. We focused on understanding the current-mediated dispersion patterns and connectivity of fish larvae in the deep-water region of the GoM by comparing several species of ecological or economic importance under different circulation scenarios, which differs from other studies focusing on a single species or a specific circulation pattern [7, 23, 101]. Future studies implementing daily particle releases during the spawning season would provide a means for examining variability in particle trajectories and connectivity between regions (e.g. [102]).

## Supporting information

S1 Table Generalized additive model (GAMs) during Season I (April to July) for target species, including only significant environmental and spatial variables. (DOCX)
