# Supplementary material for: Coupling of potential habitat models with particle tracking experiments to examine larval fish dispersal and connectivity in deep water regions
Source: PLoS One. 2024 Aug 12;19(8):e0308357. doi: 10.1371/journal.pone.0308357 (PMC11318887; doi:10.1371/journal.pone.0308357)
Supplement: S1 Table — (DOCX) [file pone.0308357.s001.docx]

**Supplementary table 1**. **GAMs during Season I (April to July) for each target species with significant environmental and spatial variables.** DE and AIC columns indicates the model’s final values without the variable. Zero (%) indicates the percentage of zero values in the response variable (larval density). Edited from Daudén-Bengoa et al. 2023.

| ***Auxis* spp.** | | | ***Notolychnus valdiviae*** | | |
| --- | --- | --- | --- | --- | --- |
| **SEASON I** | Zero (%): 63 |  | **SEASON I** | Zero (%): 18 |  |
| ***power = 1.30*** | **DE = 47.3%** | **AIC = 421.03** | ***power =1.37*** | **DE = 44.4%** | **AIC = 1119.96** |
| Depth |  |  | Depth |  |  |
| Stratification |  |  | Stratification | 41.50 | 1123.74 |
| Salinity |  |  | Salinity |  |  |
| SST | 33.5 | 438.80 | SST | 39.60 | 1126.85 |
| SSH | 43.7 | 424.86 | SSH |  |  |
| Chl | 41.6 | 427.04 | Chl |  |  |
| Wind speed | 35.4 | 433.90 | Wind speed | 37.20 | 1133.59 |
| Lat, Lon |  |  | Lat, Lon | 24.80 | 1142.28 |
| ***Bregmaceros atlanticus*** | | | ***Cubiceps pauciradiatus*** | | |
| **SEASON I** | Zero (%): 53 |  | **SEASON I** | Zero (%): 50 |  |
| ***power = 1.22*** | **DE = 43.7%** | **AIC = 551.89** | ***power = 1.38*** | **DE = 45.6%** | **AIC = 686.81** |
| Depth |  |  | Depth | 35.70 | 700.77 |
| Stratification | 33.40 | 570.24 | Stratification | 40.40 | 692.78 |
| Salinity |  |  | Salinity |  |  |
| SST | 36.90 | 567.02 | SST | 34.90 | 704.81 |
| SSH |  |  | SSH | 37.70 | 697.77 |
| Chl | 41.80 | 559.86 | Chl | 40.20 | 693.01 |
| Wind speed |  |  | Wind speed |  |  |
| Lat, Lon | 18.90 | 566.68 | Lat, Lon |  |  |
